# A comprehensive characterization of thiophosgene in the solid state

**DOI:** 10.1107/S2052520624007583

**Published:** 2024-09-05

**Authors:** Frank Tambornino, Sven Ringelband, Stewart F. Parker, Christopher M. Howard, Dominic Fortes

**Affiliations:** a Department of Chemistry Philipps University Marburg Marburg Germany; b ISIS Pulsed Neutron and Muon Facility STFC Rutherford Appleton Laboratory ChiltonOX11 0QX United Kingdom; SIMaP, France

**Keywords:** thiophosgene, X-ray diffraction, neutron powder diffraction, inelastic neutron scattering

## Abstract

Thio­phosgene is one of the principal C=S building blocks in synthetic chemistry. At room temperature, thio­phosgene is a red liquid. While its properties in the liquid and gaseous states are well known, a comprehensive characterization of thio­phosgene in its solid state is presented here. Differential scanning calorimetry shows that thio­phosgene forms a supercooled melt before rapidly crystallizing. Its melting point is 231.85 K (−41.3 °C). At 80 K, thio­phosgene crystallizes in space group *P*6_3_/*m* [No. 174, *a* = *b* = 5.9645 (2), *c* = 6.2835 (3) Å, *V* = 193.59 (2) Å^3^]. The molecule shows a distinct rotational disorder: all S and Cl positions are of mixed occupancy and the disorder does not resolve at temperatures as low as 10 K, as was shown by neutron powder diffraction. Infrared, Raman and inelastic neutron scattering spectra were collected and assigned with the aid of quantum chemical calculations. A larger ordered structural model allowed for better agreement between the measured and calculated spectra, further indicating that disorder is an inherent feature of solid-state thio­phosgene.

## Abstract 2

Thio­phosgene crystallizes in space group *P*6_3_/*m* with two rotationally disordered molecules in the unit cell. The IR, Raman and inelastic neutron scattering spectra of solid thiophosgene are reported for the first time.

## 1. Introduction

When it comes to malodorous compounds, sulfur-containing molecules are often at the forefront. Commonly referred to as the ‘rotten egg’ smell, the odour of hydrogen sulfide, H_2_S, is widely known. Although it exhibits not only an unpleasant smell, but is also highly toxic, H_2_S is an important chemical as precursor to elemental sulfur and a wide range of sulfur-containing substances. Other notable sulfur-containing compounds are, for example, allicin (giving garlic its characteristic taste and smell), 2-mercapto­ethanol (a nucleophile in organic synthesis), tetra­hydro­thio­phene (used as odorant in natural gas and used as solvent in niche applications) and thio­acetone which is sometimes referred to as the ‘most malodorous compound in the world’. Nevertheless, many such compounds are standard chemicals in industry where they are produced and used on a multi-tonne scale per year.

One of the most important and versatile sulfur-based intermediates and C=S building blocks in the chemical industry is thio­phosgene. It is a key reagent in various chemical reactions and finds extensive application in the pharmaceutical industry for the synthesis of drugs. In polymer science it is a precursor in the manufacture of iso­thio­cyanates and it is also commonly used in the production of pesticides, dyes and other fine chemical compounds. In research laboratories it is sometimes used as a substitute for phosgene as thio­phosgene is slightly less toxic and easier to handle.

Thio­phosgene was first synthesized in 1843 by the reaction of elemental chlorine with carbon di­sulfide, however, it is debated whether a pure substance was isolated as opposed to a mixture of carbon di­sulfide and carbon tetrachloride (Kolbe, 1843 ▸; Tilles, 1966 ▸). In 1887 it was shown that thio­phosgene can conveniently be synthesized by reduction of tri­chloro­methane­sulfenyl chloride with zinc, and this method is still prevailing in today’s processes (Klason, 1887 ▸).

At room temperature, thio­phosgene is a red liquid which fumes lightly in contact with air. When highly diluted, it has a sweet smell, but in just slightly higher concentrations it is severely malodorous (also described as pungent, disagreeable, suffocating) with a very distinctive smell. Despite this, it is commonly used in industry and research laboratories around the world and as such many key properties have been elucidated.

Thio­phosgene has a boiling point of 346.15 K (73 °C) and a density of 1.5085 g cm^−3^ at 288 K (Billeter & Strohl, 1888 ▸). Its vibrational modes have been studied by infrared and Raman spectroscopy in liquid and in solution (Lowell & Jones, 1960 ▸; Burnelle, 1956 ▸; Tilles, 1966 ▸; Jones *et al.*, 1957 ▸). The molecule exhibits a trigonal planar geometry. From gas-phase electron diffraction, the C=S bond length was determined to be 1.602 (5) Å and the C—Cl bond length to 1.72 8 (3) Å with a Cl—C—Cl angle of 111.2 (3)° (Nakata *et al.*, 1982 ▸). It has been calculated that the C—Cl bond exhibits a 12% double bond character which is lower than in its lighter chalcogen homologue phosgene (COCl_2_), which shows a 17% double bond character (Tilles, 1966 ▸). Notably absent are examinations of thio­phosgene in the condensed phase. Neither the solidification or melting temperatures, nor its crystal structure have been reported. Vibrational spectra of solid thio­phosgene are also not reported.

In this work we present an in-depth study of solid state thio­phosgene. We report its crystal structure as determined from both single crystal X-ray and variable temperature neutron powder diffraction, its melting point from low temperature differential calorimetry data and its IR, Raman and inelastic neutron scattering spectra including a full assignment of modes. We find that the substance crystallizes in hexagonal space group *P*6_3_/*m* with rotational disorder that does not resolve even at temperatures as low as 10 K.

## 2. Results and discussion

### 2.1. Crystal structure

#### 2.1.1. Single crystal X-ray diffraction

A small amount of thio­phosgene was condensed into a glass capillary (diameter 0.3 mm, Lindemann Spezialglas) which was subsequently flame-sealed under vacuum. It was then centred on the goniometer of a StadiVari Diffractometer (Stoe & Cie GmbH, Darmstadt, Germany), equipped with an Oxford cryostream set to room temperature. Upon cooling at 10 K min^−1^ the sample rapidly solidified at ∼210 K (−63 °C), indicative of solidification from a supercooled melt. Due to the low crystallinity, during a test measurement no diffraction spots were observed. The sample was carefully heated in 1 K increments until partial melting of the sample was observed, which commenced at ∼240 K (−33 °C). A sinusoidal temperature profile oscillating ±0.5 K around 240 K (−33 °C) was applied to grow a single crystal through Ostwald ripening. After one hour, test measurements confirmed the growth of a single crystal in the hexagonal system (low Laue class) with unit-cell parameters *a* = *b* = 6.0132 (6) and *c* = 6.513 (9) Å. The specimen was cooled to 235 K (−38 °C) before a full dataset was collected. After the measurement, the specimen was subsequently cooled to 80 K (−193 °C) to collect a second dataset.

**Figure 1:**
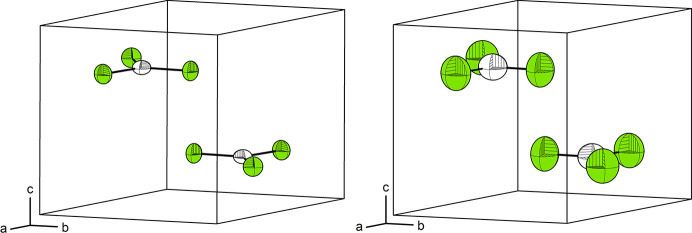
Crystal structure of thio­phosgene as deduced from X-ray diffraction at 80 K (left) and 235 K (right). Colour code: White: carbon, green: mixed S/Cl occupancy. Displacement ellipsoids are drawn at a 75% probability level.

Thio­phosgene crystallizes in the hexagonal crystal system with space group *P*6_3_/*m* [No. 174, *a* = *b* = 5.9645 (2), *c* = 6.2835 (3) Å, *V* = 193.59 (2) Å^3^ at 80 K; *a* = *b* = 6.0132 (6), *c* = 6.5130 (9) Å, *V* = 203.95 (5) Å^3^ at 235 K] with two formula units in the unit cell (see Fig. 1 ▸). The carbon atom occupies Wyckoff position 2*c* (site symmetry 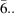), around which the sulfur and chlorine atoms form a regular triangle and occupy the same Wyckoff position 6*h* (site symmetry *m*..). From Fourier maps, no secondary maxima in electron density can be discerned. Thus, the molecule shows rotational disorder about the carbon atom resulting in the Cl/S site having a two-thirds occupancy by chlorine and one-third with sulfur. The C—S/Cl interatomic distance is 1.6806 (8) Å and 1.6748 (14) Å at 80 K and 235 K, respectively. Our value as determined from X-ray diffraction lies in the middle between the distances as determined from gas-phase electron diffraction {C—Cl [1.728 (3) Å] and C=S [1.602 (5) Å]}, further supporting a model with mixed occupancy.

As the X-ray scattering lengths of S and Cl differ only by about 6%, an unequivocal assignment with X-ray diffraction is difficult. Starting from our model, resolving the disorder is only possible through a reduction of the symmetry by either descending to a lower symmetry space group (*translationengleiche* reduction) or enlargement of the unit cell (*klassengleiche* reduction), with the latter accompanied by superstructure reflections. We tested both possibilities. If the structure is re-evaluated and calculated in *P*1 (No. 1), the same model results, including the disorder. Careful examination of calculated precession images did not reveal any superstructure reflections, further indicating a correct assignment of the data. The model is further corroborated by neutron powder diffraction, see below.

**Figure 2:**
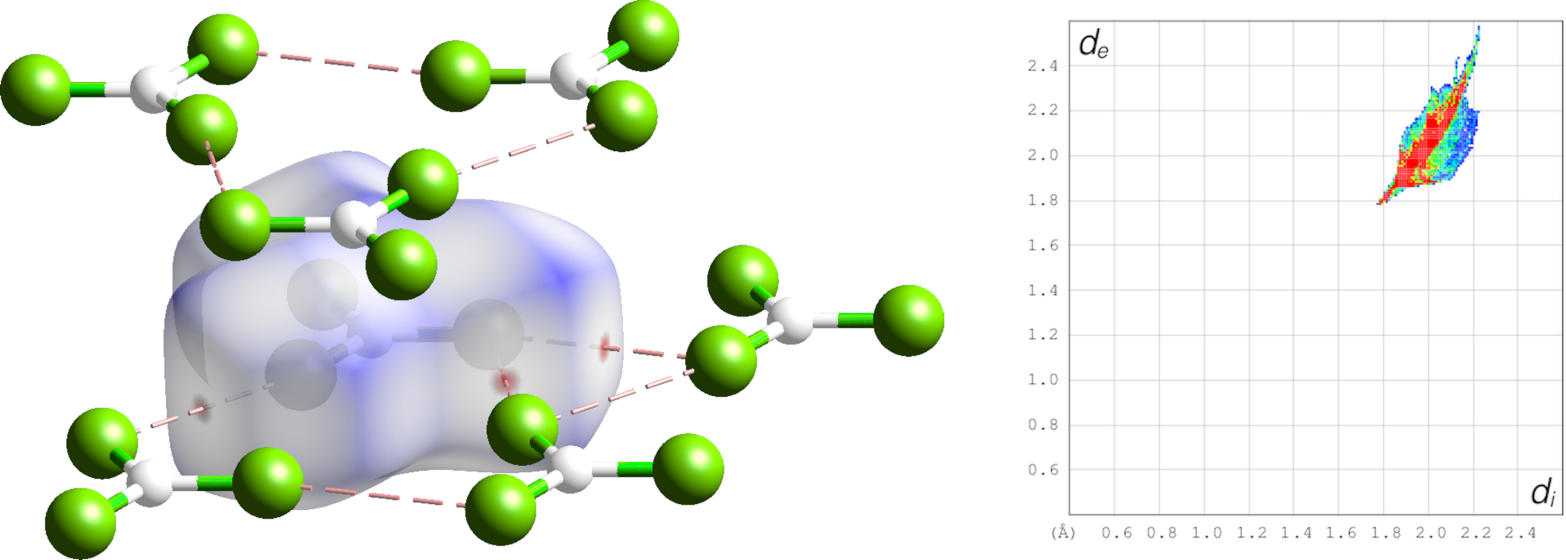
Hirshfeld surface (left) and fingerprint plot (right) for thio­phosgene at 80 K. Colour code: white: carbon, green: mixed S/Cl occupancy, short contacts indicated as pale-red dashes.

The barycentres of the individual molecules correspond with a hexagonal close packing motif. Compared with the *c*/*a* ratio of 1.633 for an ideal hexagonal closest packing, the ratio in thio­phosgene is only *c*/*a* = 1.0535. This shortening of the *c* axis in comparison to the *a* axis is due to the anisotropy of the molecules. Hirshfeld surface analysis (see Fig. 2 ▸) reveals weak intermolecular interactions solely in the {0001} planes. Every thio­phosgene molecule exhibits six short contacts from the terminal S/Cl positions to the neighbouring molecules with interatomic distance 3.5672 (8) Å (at 80 K), which is just within the range of the sum of their van der Waals radii [*r*_vdW_(S) = 1.80 Å, *r*_vdW_(Cl) = 1.75 Å]. Accordingly, the fingerprint plot is very sharp with only a small spike for said contacts (see Fig. 2 ▸, right).

In contrast to the disordered model of thio­phosgene, the crystal structure of its lighter chalcogen homologue phosgene (COCl_2_) is fully ordered (Zaslow *et al.*, 1952 ▸). Phosgene crystallizes in the tetragonal system with space group *I*4_1_/*a* [No. 88, *a* = *b* = 15.82 (5), *c* = 5.72 (2) Å, at 113.15 K]. Interestingly, there is no relationship between the crystal structures of phosgene and thio­phosgene. They are neither related by a direct group-subgroup relation, nor do they share a common supergroup of any index.

#### 2.1.2. Neutron powder diffraction

The X-ray scattering lengths of S and Cl differ only by 6% which is lower than the commonly used threshold of 10% that would allow for a definite assignment of the atom types. For neutron diffraction the scattering lengths of S and Cl are 2.8 and 9.6 fm, respectively, allowing for unambiguous differentiation. Thus, we address the question of whether an ordering can be observed with neutron powder diffraction (Tambornino *et al.*, 2024 ▸). The measurements were performed at temperatures as low as 10 K so that any temperature-dependent ordering phenomena could be observed.

To prepare the sample for neutron diffraction, thio­phosgene was added dropwise into a steel mortar which was filled with liquid nitro­gen. The shock-frozen sample was finely ground under liquid nitro­gen, transferred to a pre-cooled aluminium slab-geometry container (slab can, internal dimensions 18 × 23 mm perpendicular to the incident neutron beam and 10 mm deep parallel to the beam), held in place by vanadium foil windows on the beam in/out faces that were sealed with indium wire. Exposed components of the cell were masked with Gd and Cd foils to prevent unwanted coherent scattering from the Al and steel components of the slab can. For accurate and precise temperature control a 30 mm cartridge heater was inserted into the aluminium frame of the sample holder slab along with a RhFe resistance thermometer. The assembly was transferred into a closed-cycle refrigerator (CCR) held at 100 K.

Data were collected on the High Resolution Powder Diffractometer (HRPD) at the ISIS neutron spallation source using the instrument’s standard 30–130 ms time-of-flight (TOF) measurement window. HRPD has detector banks in backscattering geometry (2θ = 158–176°), at 90° to the incident beam (2θ = 80–100°) and in forward scattering (2θ = 28–32°), which provide *d*-spacing coverage in the 30–130 ms TOF window from 0.65–2.60, 0.85–3.90 and 2.3–10.2 Å, respectively. The measurements were performed in 10 K increments on cooling initially from 100 K down to 10 K, and then on heating from 110 K up to 240 K. Longer integrations suitable for high quality structure refinements were acquired at 10 K (4 h) and at 50, 100, 150 and 200 K (2 h each). All other points were measured for ∼25 min with the aim of obtaining only precise unit-cell parameters. However, it proved possible to obtain good structure refinements even from the shorter measurements. Between each datum, the temperatures were changed at 3 K min^−1^ and a period of 10 min was allowed for thermal equilibration prior to the start of the measurements.

The diffraction data were time-focused, normalized to the incident spectrum and corrected for instrument efficiency by reference to a V:Nb null-scattering standard using *Mantid* (Arnold *et al.*, 2014 ▸) and exported in a format suitable for analysis using standard Rietveld refinement codes.

#### 2.1.3. Refinement methodology and variable-temperature measurements

**Figure 3:**
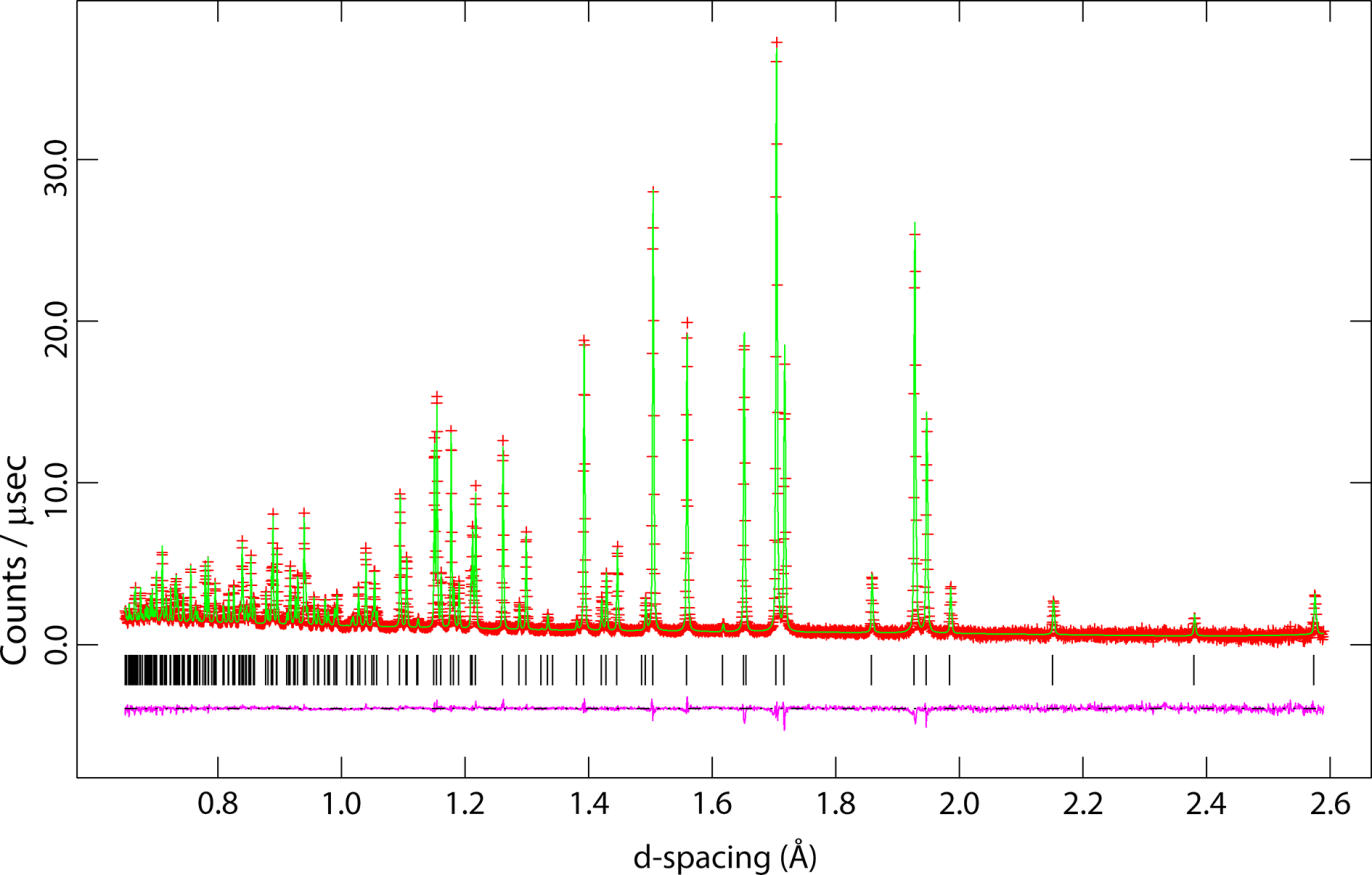
Results of the Rietveld refinement of thio­phosgene at 10 K. Red crosses indicate measured data, green line the refined model and purple line the difference; Bragg peak markers are shown as black vertical lines under the diffraction pattern.

The neutron powder diffraction data were refined at 10 K by the Rietveld method using *GSAS/Expgui* (Larson & Von Dreele, 2004 ▸; Toby, 2001 ▸) starting from the structure established using single-crystal XRD (see above). Data were fitted by refinement of the unit-cell parameters, scale factor, peak-profile parameters (*GSAS* profile function #3), and a six-term shifted-Chebyshev polynomial. Atomic coordinates were refined independently without restraints, as were the occupancies of the S and Cl and anisotropic displacement parameters, and *U_ij_* of all atoms. It was found necessary to refine a sample texture correction using a sixth-order spherical harmonic model. The final fit to the data at 10 K has *R*_wp_ = 3.84%; the fit to the data is shown in Fig. 3 ▸. Occupancy factors for the S and Cl refined freely to 0.338 (5) and 0.662 (5), respectively. Since these values are statistically insignificantly different from one-third and two-thirds and since refinement of these occupancies led to a higher than desirable value for the ratio of the least-squares parameter shift to the standard uncertainty, it was decided to fix the occupancies at one-third and two-thirds for all subsequent refinements. The refined structural parameters are given in an electronic supplementary crystallographic information file (CIF).

Refinements at each temperature step using the Rietveld method included changes to the unit-cell parameters, atomic coordinates (which were freely refined), thermal displacement parameters (set anisotropic and freely refined), the background, scale factor, and peak profile parameters; as noted above, the S/Cl occupancies were fixed.

Rietveld refinement of a measurement performed at 10 K (see Fig. 3 ▸), corroborated the crystal structure model as deduced from X-ray diffraction. Neither superstructure reflections, nor additional reflections indicating symmetry reduction were observed. Solely a contraction of the unit cell due to thermal effects is evident.

**Figure 4:**
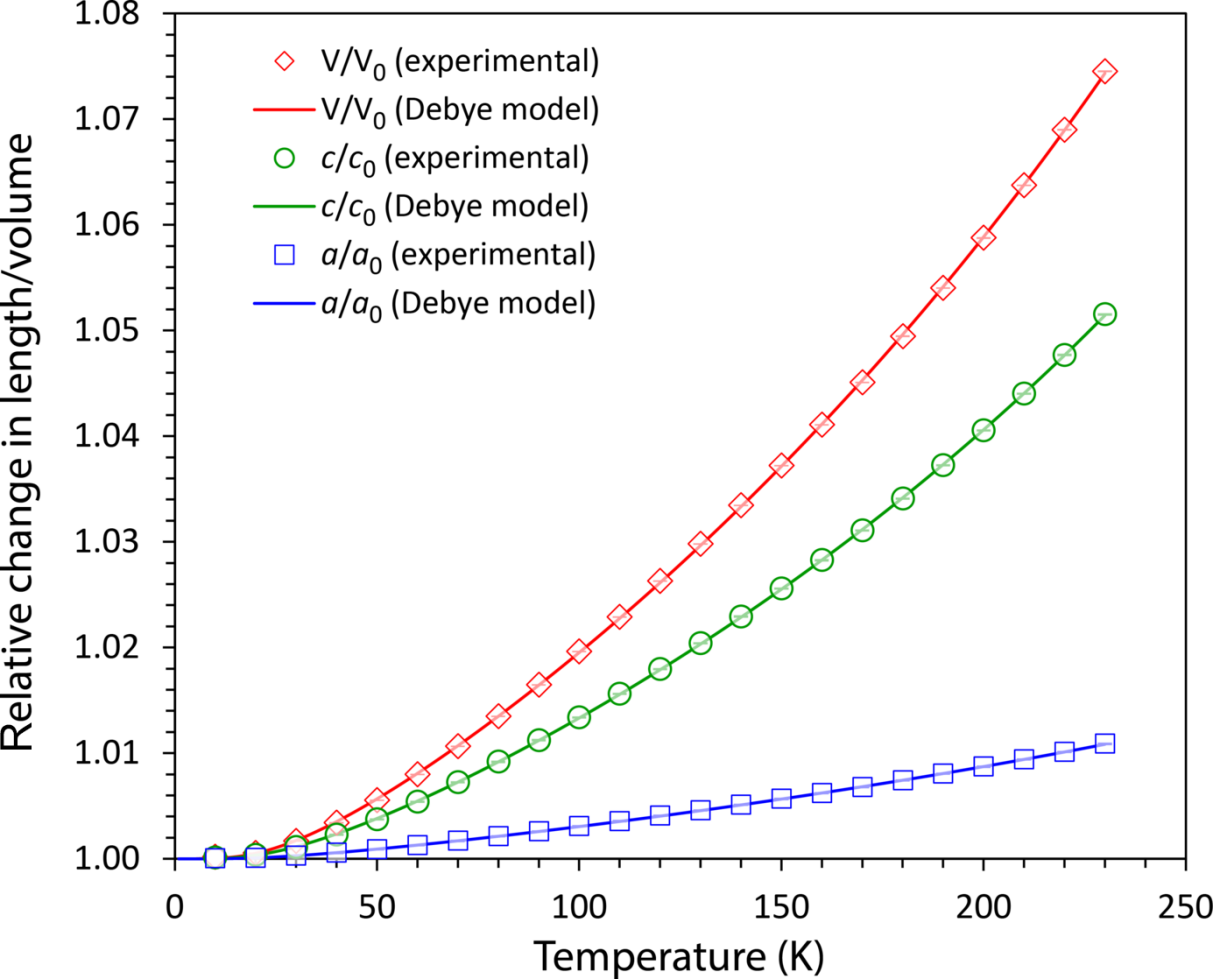
Relative variation in the unit-cell parameters of thio­phosgene at different temperatures. Symbols denote values resulting from Rietveld refinements (error bars are substantially smaller than the symbols); the solid lines are obtained from the fitting of a Debye-type model to the unit-cell parameters, as described in the text. The absolute values of the unit-cell parameters are tabulated in Table S1 and plotted in Figs. S1, S2 and S3.

The variable temperature measurements allowed for the extraction of unit-cell parameters in 10 K steps between 10 and 230 K (see Fig. 4 ▸). Both the *a* and *c* unit-cell parameters enlarge with increasing temperature. However, the overall expansion of the *a* axis is considerably less than that of the *c* axis (+1.084 and +5.141%, respectively); the total volume change from 10 K to 230 K is +7.434%. The explanation for this anisotropy of the thermal expansion lies in the intermolecular interactions. In {0001} the molecules are held together more strongly than in the [1000] direction, which is concomitant with the observation from Hirshfeld analysis.

**Figure 5:**
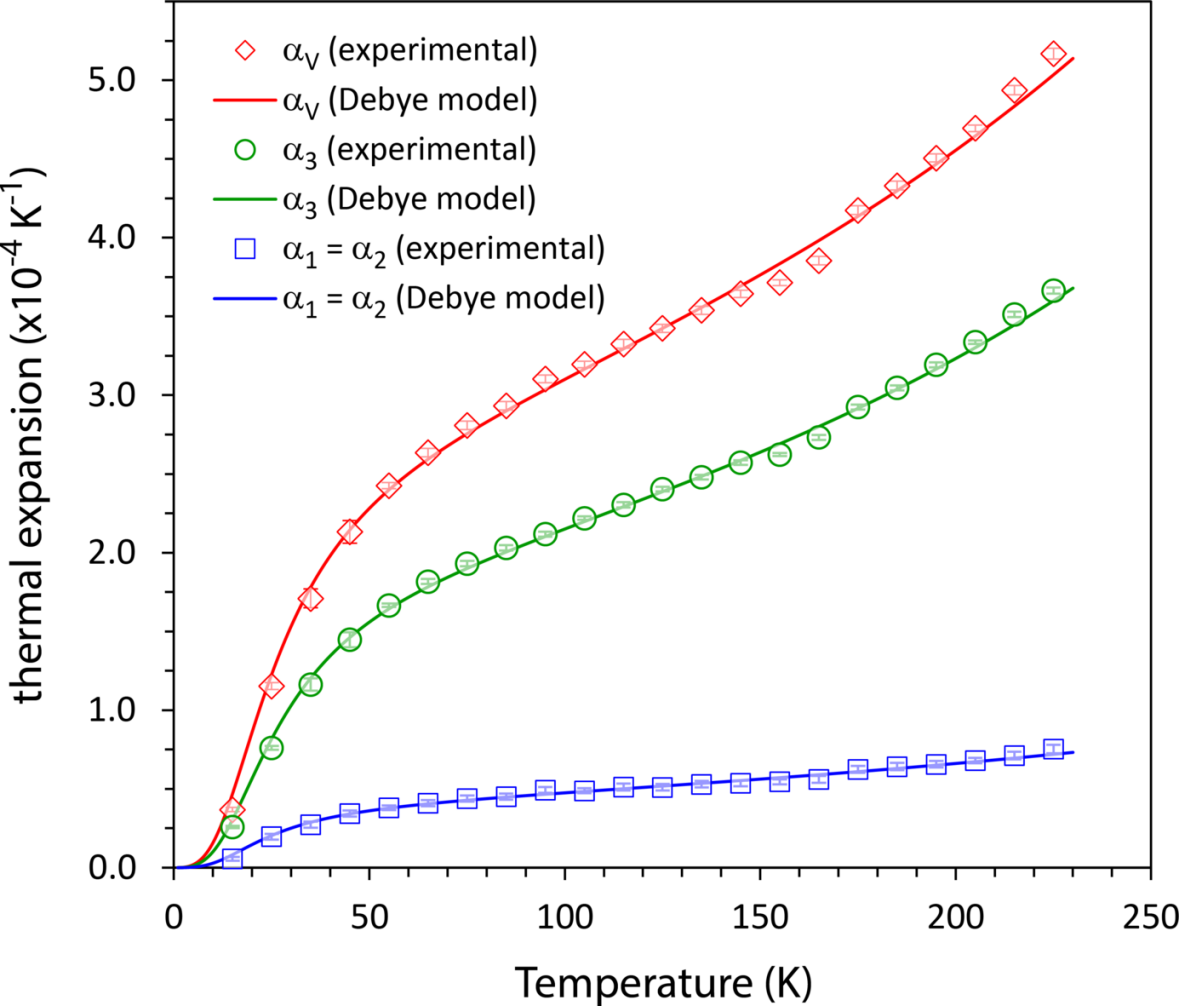
Coefficients of linear thermal expansion along [1000] and [0001] (α_1_ and α_3_, respectively) and the volume thermal expansion (α_*V*_) as a function of temperature in thio­phosgene. Symbols denote values obtained from point-by-point derivatives of the refined unit-cell parameters with respect to temperature and the solid line corresponds with the thermal expansion found from fitting a Debye-type model to the unit-cell parameters (see text for details). The values reported here are tabulated in Table S2 and plotted separately in Figs. S4, S5 and S6.

Eulerian infinitesimal strain tensors were calculated from pairs of unit-cell parameters determined at adjacent temperatures and then normalized by the temperature increment between them in order to obtain thermal expansion tensors, *i.e*. unit-strain tensors. Standard matrix decomposition methods were used to derive the eigenvalues and eigenvectors of the thermal expansion tensor, these being the magnitudes and orientations of the principal expansivities, although in this instance the orientations are fixed by the symmetry of the crystal. The temperature dependences of these principal linear expansivities, α_1_ = α_2_, α_3_ and the volume thermal expansion, α_V_ are shown in Fig. 5 ▸.

The unit-cell parameters of thio­phosgene have been fitted with a second-order Grüneisen approximation to the zero-pressure equation of state (Cochran, 1973 ▸). In this approximation, the thermal expansion is considered equivalent to elastic strain such that,
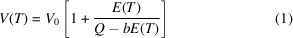
where *V*_0_ is the unit-cell volume at zero pressure, *b* = ½ (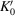 −1) and *Q* = (*V*_0_*K*_0_/γ); *K*_0_ is the zero-pressure isothermal bulk modulus, 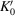 is its first derivative with respect to pressure, and γ is the thermal Grüneisen parameter. The internal energy due to lattice vibrations, *E*(*T*), is then estimated via a simple Debye model approximation of the phonon density of states:
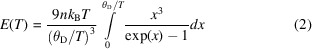
where θ_D_ is the Debye temperature, *n* is the number of atoms per molecule, and *k*_B_ is the Boltzmann constant; the integral term is evaluated numerically. For the purpose of using equation (1) to model the unit-cell parameters of the crystal, whilst remaining dimensionally correct, the lengths of the *a* axis and *c* axis as a function of temperature have been fitted as *a*^3^ and *c*^3^ respectively. Table 1 ▸ reports the parameters obtained from fitting equation (1) to the unit-cell parameters of thio­phosgene.

The location of the Debye cut-off is consistent with the high-frequency edge of the external vibrational modes at ∼80 cm^−1^ observed by inelastic neutron spectroscopy (INS), described later, and the statistically insignificant difference in cut-off between the *a* axis and *c* axis fitting also agrees with the calculation reported below indicating a very small vibrational dispersion.

Of some interest is the apparent discontinuity in the thermal expansion curves at 170 K; the appearance of this feature is very similar to that observed in shock-frozen water ice, which has been interpreted as the annealing out of defects, introduced during the rapid solidification (Fortes, 2019 ▸).

When heated above 230 K, the intensity of the observed Bragg peaks drops, which we attribute to the onset of melting. At 240 K, the sample was observed to be entirely melted, so the temperature was reduced back to 230 K and several short datasets (8 min) were then collected on heating in 1 K increments. Evidence of pronounced melting was apparent at 233 K and the sample was fully molten at 234 K. The sample was cooled a second time, down to 220 K, to induce recrystallization. This is in reasonable agreement with DSC data, see below.

**Figure 6:**
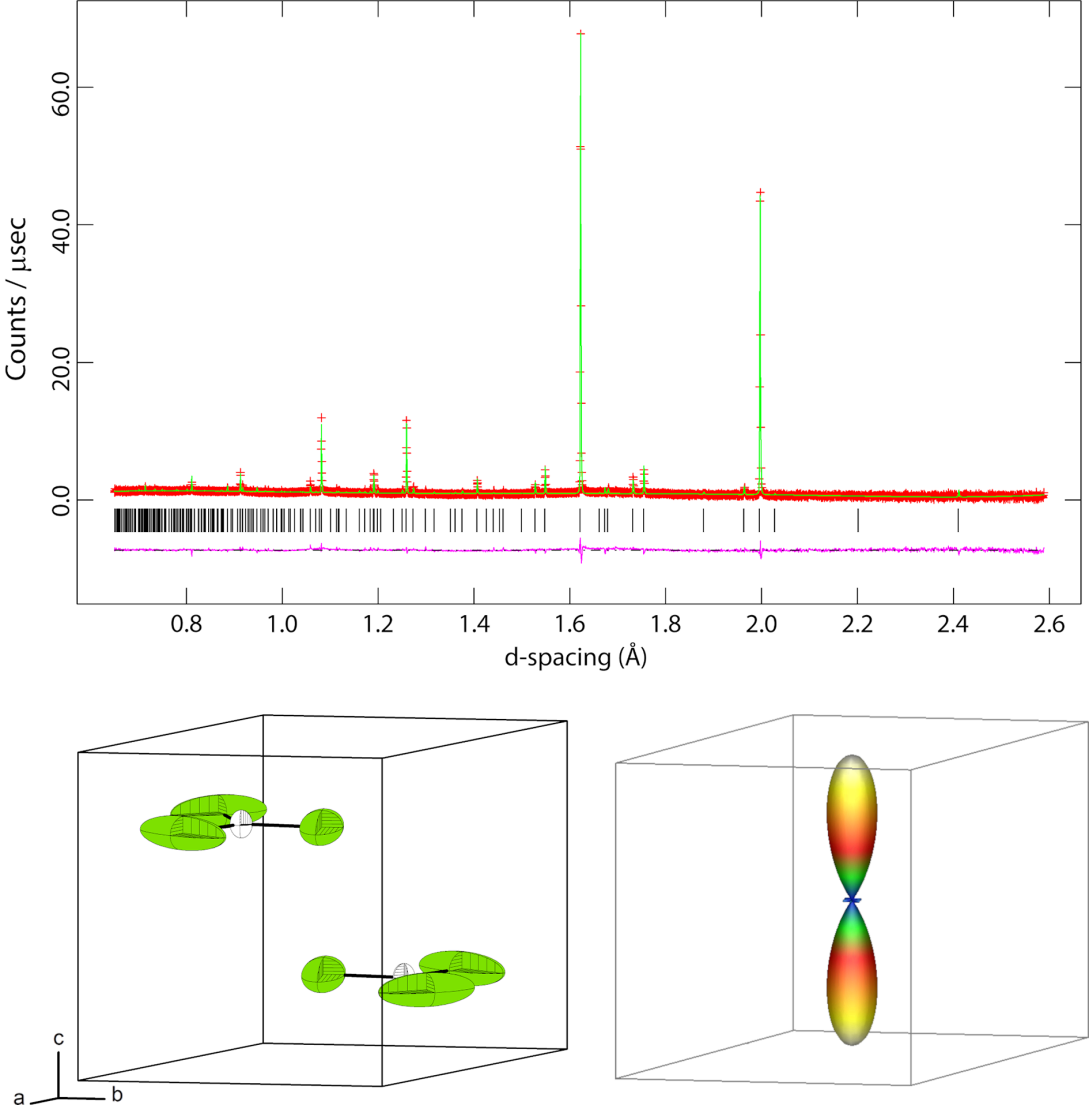
Top: Result of the Rietveld-refinement of thio­phosgene after one melting and solidification cycle. Strong preferred orientation is observed. Red crosses indicate measured data, green line the refined model and purple line the difference. Bottom left: Structural model resulting from the Rietveld refinement. The thermal displacement ellipsoids for the S/Cl position are prolate as a result of the preferred orientation. Bottom right: Three-dimensional sixth-order spherical harmonic representation of the sample showing string preferred orientation along the [0001] direction.

After melting and subsequent solidification, we collected additional diffraction data. While the reflection positions were very similar to the collected data before, the intensities were vastly different. For example, the former strongest reflection (1 1 3) was now lower in intensity and the (0 0 4) reflection became the most intense in the diffraction pattern. We attribute this to texture as the sample could have grown in larger oriented crystals upon solidification. As such, the data was modelled with a spherical harmonics function to account for the preferred orientation and the result of the Rietveld refinement can be found in Fig. 6 ▸ (Coelho, 2018 ▸). The visual three-dimensional sixth-order spherical harmonic representation of the sample’s texture supports this, see Fig. 6 ▸.

**Figure 7:**
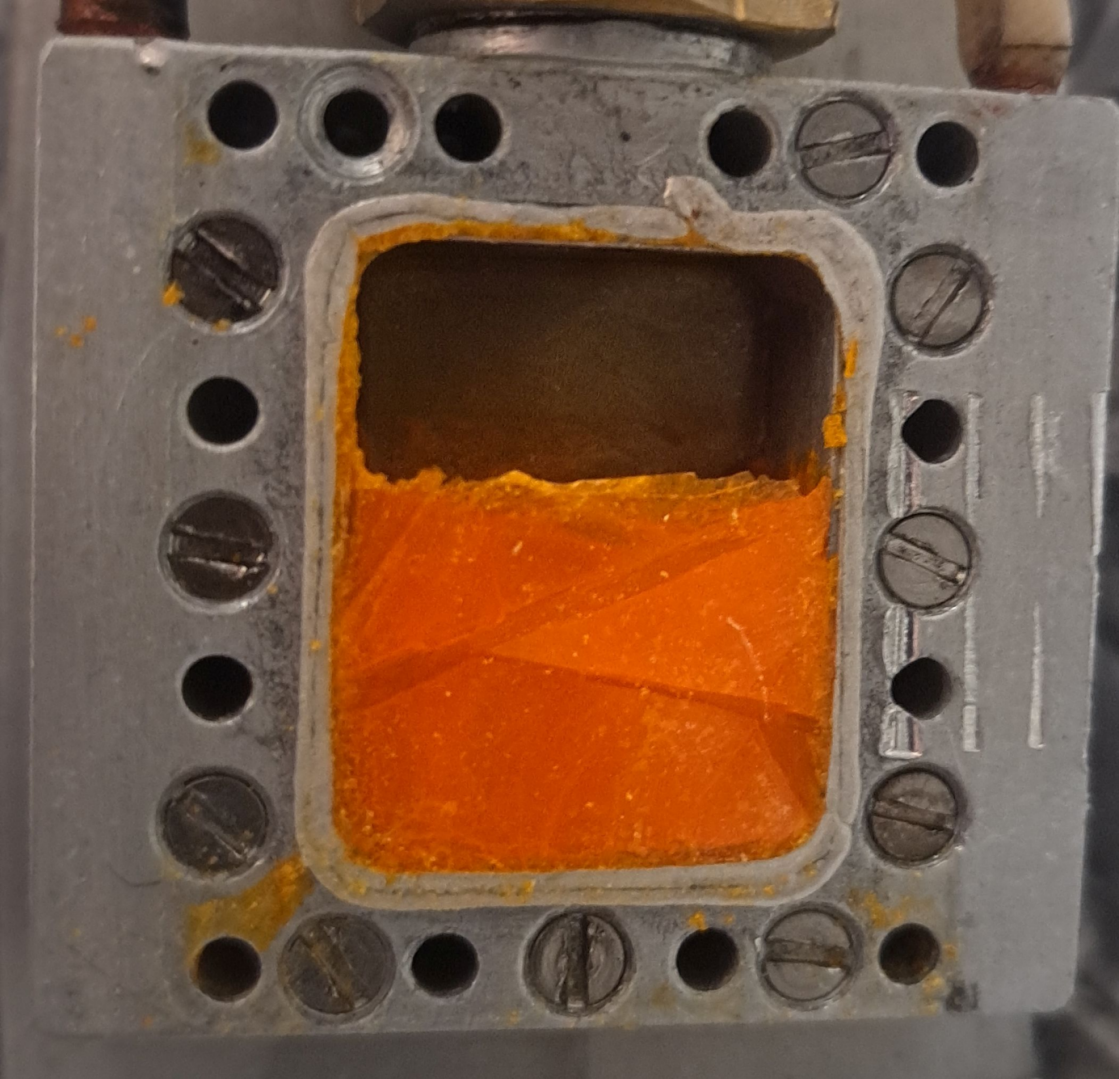
Thio­phosgene in the slab can after melting and solidification. The sample exhibits three large crystalline areas which result in strong preferred orientation of the collected ‘powder’ data.

While the determination of the unit-cell parameters [*a* = *b* = 5.9921 (2) Å, *c* = 6.4805 (2) Å, *V* = 201.51 (2) Å^3^] was in accordance with our earlier measurements, and the model is in principle similar, the anisotropic displacement parameters exhibited some particularities, being much larger. For the central C atom, the displacement parameters are in the range of the former measurement. However, the displacement ellipsoids of the Cl/S position derived from refinement of these strongly textured data are extremely prolate. Examination of the Bragg peaks with HRPD’s detectors switched into a fully pixelated mode revealed that the sample had recrystallized into a substantially single-crystal form, as the usual Debye–Scherrer arcs were transformed into streaks and spots. This was confirmed later when the sample holder was opened under liquid nitro­gen and visual inspection revealed only a few large single-crystal domains in the sample (see Fig. 7 ▸). It is a matter of interest to note that a tolerably accurate structural model could still be obtained even from a specimen of this kind.

### 2.2. Differential scanning calorimetry

During single crystal growth in the capillary, we observed supercooling and rapid solidification of the sample at around 210 K (−60 °C). Melting of the sample in this environment commenced at 240 K (−33 °C). However, those solidification and melting temperatures must be viewed with caution. Temperatures of cooling streams optimized for single-crystal diffractometers are only calibrated for a very small sample volume which is usually a small crystallite of roughly 0.001 mm^3^. Our sample was sealed in a capillary with a diameter of 0.3 mm. A larger sample volume than needed was also used to effectively grow a single crystal. Additionally, the sample in the capillary was flame-sealed under vacuum, which might have a small effect on the observed temperatures, too. More reliable are the temperature-dependent neutron powder diffraction patterns, as the cryostat is optimized for the larger sample volume and the sample can. Here, we observed half intensity of the diffraction pattern at 230 K (−43 °C), indicating that half of the sample had undergone a substantial degree of melting.

To accurately measure the melting and solidification points, and the corresponding heats of melting and crystallization, we performed low temperature differential scanning calorimetry. The measurements were performed on a heat flow differential scanning calorimeter model STARe System DSC 3 (Mettler Toledo, Columbus, Ohio, United States). A constant stream of nitro­gen (10 cm^3^ min^−1^) was used as purging gas.

**Figure 8:**
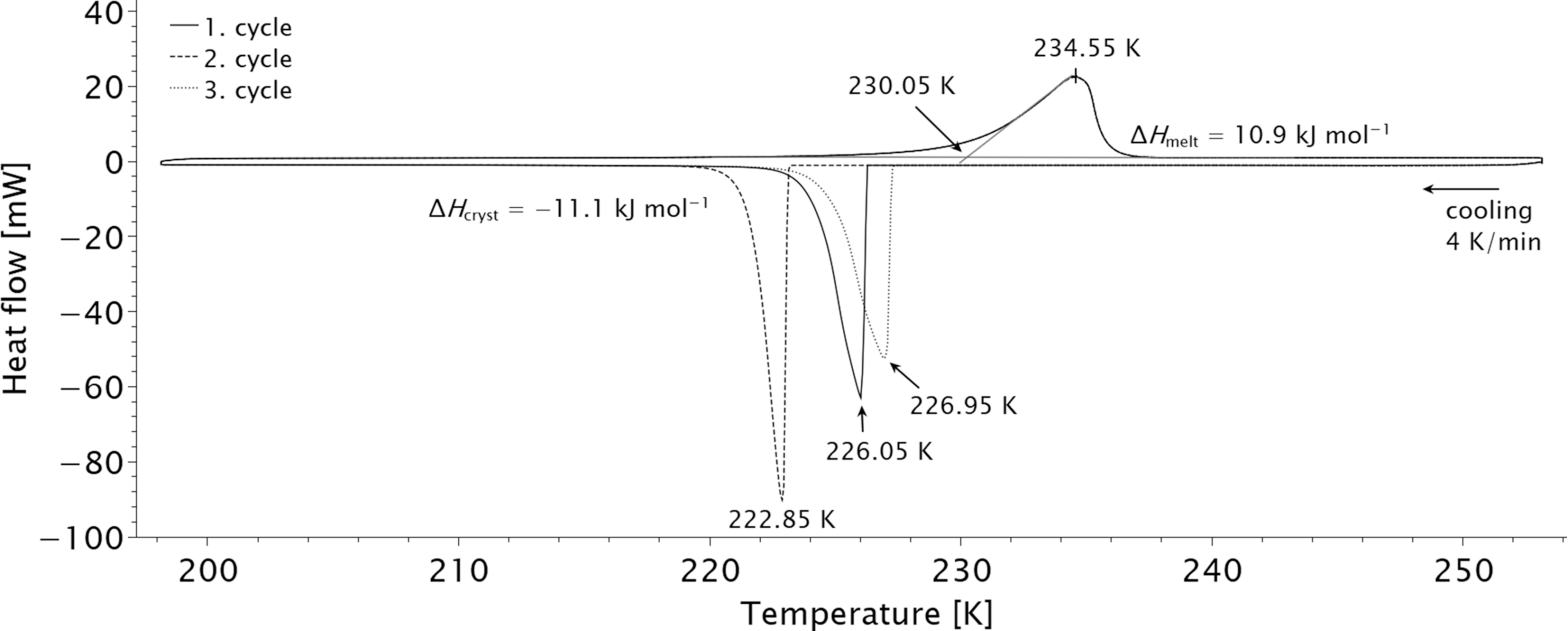
Differential scanning calorimetry data of thio­phosgene. Three subsequent measurements were performed which differ in their respective solidification temperatures. The curves for the melting temperature coincide. For details of the fit see text.

Thio­phosgene (14.46 mg, 0.126 mmol) was placed in a 40 µL aluminium crucible with a pin profile which was subsequently closed with a press. The temperature program was run for three cycles from 198.15 K to 253.15 K using a cooling and heating rate of 4 K min^−1^. The data was evaluated using the *STARe* program (Mettler Toledo). The extrapolated onset-melting-temperature was defined by the intersection point of the extrapolated baseline (green, see Fig. 8 ▸) and the inflectional tangent (pink, see Fig. 8 ▸) at the beginning of the melting peak. The corresponding melting enthalpy was determined by the absolute integral and the weighted sample (J g^−1^) of the heat flow signal between 218.15 and 243.15 K and converted into kJ mol^−1^. During the three cycles we found the following values for the melting temperature and the melting enthalpies: 230.15 K (10.9 kJ mol^−1^), 230.05 K (10.9 kJ mol^−1^) and 230.05 K (11.0 kJ mol^−1^). The crystallization peak temperature and corresponding enthalpies are 226.05 K (11.0 kJ mol^−1^), 222.85 K (11.2 kJ mol^−1^) and 226.95 K (11.1 kJ mol^−1^).

During cooling, we found that a supercooled melt can be obtained with peak solidification temperatures up to 7 K lower than the melting temperatures. Regardless of the measurement cycle, the onset of the signal for melting, and thus the melting point, is similar. The melting point of thio­phosgene is 231.85 K (−43.1 °C) and experimentally derived values for the latent heat of crystallization and melting are similar at ∼11 kJ mol^−1^.

### 2.3. Vibrational spectroscopy

**Figure 9:**
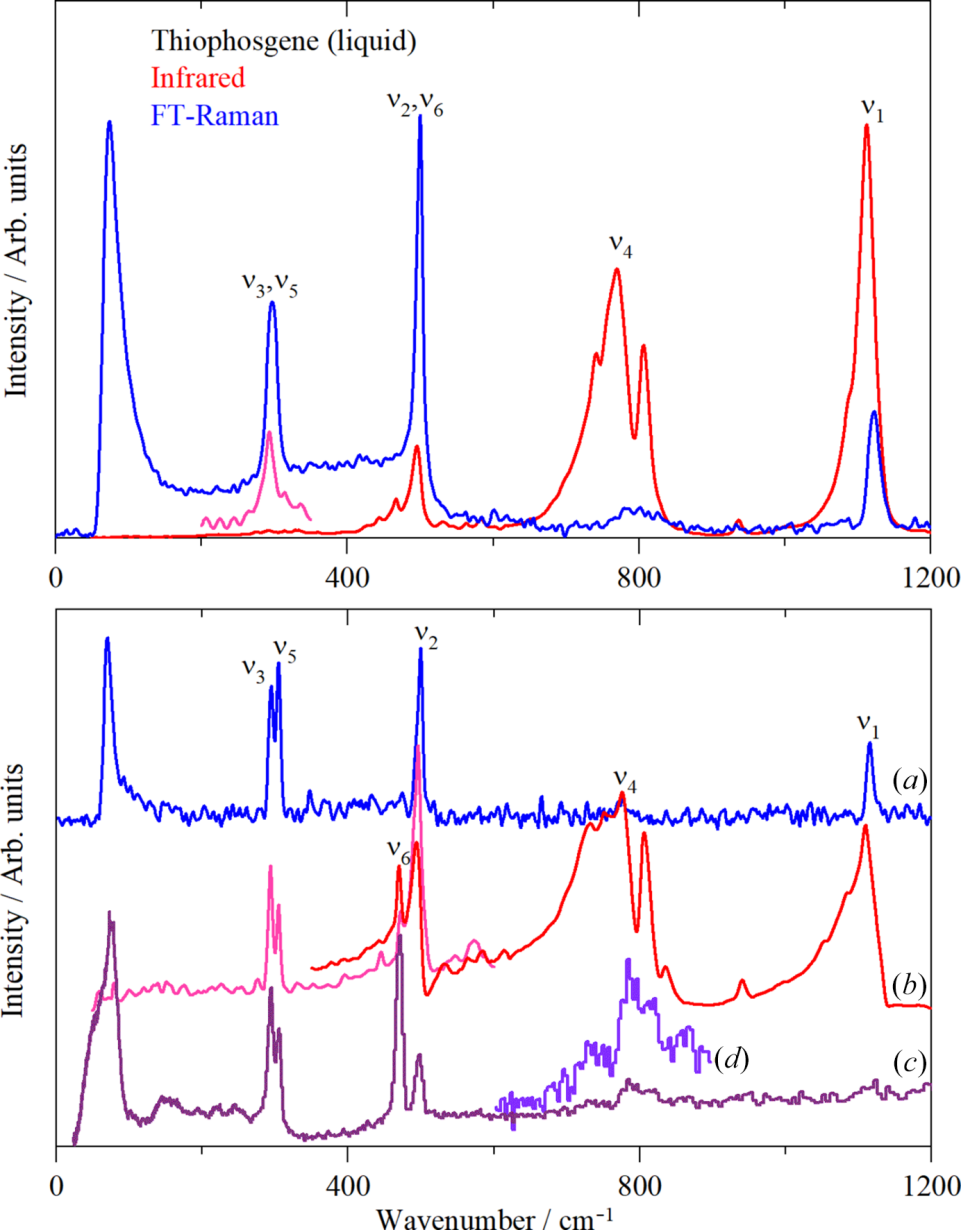
Vibrational spectra of thio­phosgene showing the assignments. Top: liquid phase at room temperature. Bottom: solid state. Trace (*a*) FT–Raman at ∼200 K, trace (*b*) infrared at ∼200 K (red is by ATR and pink by transmission), trace (*c*) INS at ∼10 K and trace (*d*) 4 × ordinate expansion of (*c*).

In the gas and liquid phases thio­phosgene has *mm*2 symmetry and the four atoms give rise to six modes: C=S stretch (ν_1_), C—Cl symmetric (ν_2_) and antisymmetric stretch (ν_4_), S=C—Cl symmetric (ν_3_) and antisymmetric in-plane bend (ν_5_) and an out-of-plane bend (ν_6_). As might be expected for a simple molecule, the spectroscopy in the gas and liquid phases has been comprehensively studied (Lowell & Jones, 1960 ▸; Burnelle, 1956 ▸; Tilles, 1966 ▸; Jones *et al.*, 1957 ▸). Our liquid phase spectra, Fig. 9 ▸ top part, are in complete agreement with the literature spectra, the assignments of ν_1_ to ν_6_ are indicated in the figure. It can be seen that ν_3_ and ν_5_ are very close in energy as are ν_2_ and ν_6_.

The solid state has been much less studied, probably because of the absence of a crystal structure model. Fig. 9 ▸ lower part shows the Raman, infrared and inelastic neutron scattering (INS) spectra of the solid state. The major differences between the liquid and solid states are that the bands at ∼300 and 500 cm^−1^ are clearly split and the other bands show more structure. There are several possible reasons for these effects: the sharper bands in the solid state allow the individual bands to be resolved including the ^35/37^Cl isotope splitting and the presence of more than one molecule in the primitive cell results in factor group splitting. It is also apparent from the INS spectrum that the external modes (the translations and librations in the 0–200 cm^−1^ region) are all very close in energy. This is understandable as S and 2 × Cl account for ∼90% of the mass of the molecule, thus the energy required to rotate (librate) will be similar to that needed to translate it.

**Figure 10:**
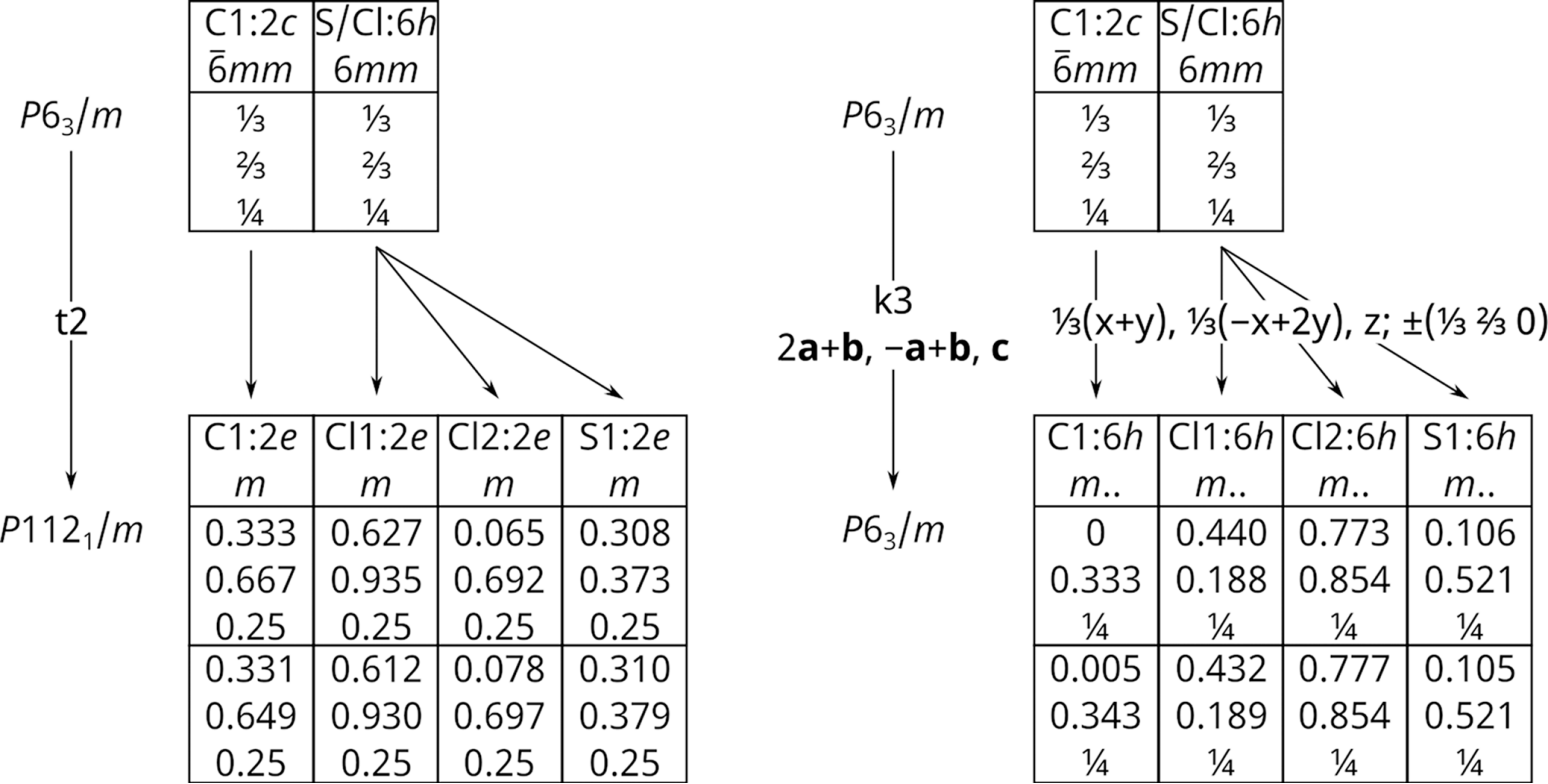
Schemes for the symmetry reduction (Bärnighausen tree) of the disordered crystal structure of thio­phosgene to facilitate quantum mechanical calculations. Left: *Translationengleiche* symmetry reduction leading to a model in the non-standard setting *P*112_1_/*m* (No. 11, standard setting *P*12_1_/*m*1) with *Z* = 2. Right: *Klassengleiche* symmetry reduction leading to an enlarged unit cell in *P*6_3_/*m* with *Z* = 6. Optimized coordinates from quantum chemical calculations are added in the bottom row.

To understand which, or all, of these are responsible for the spectra, computational studies are required. However, these are complicated by the disorder. Vibrational spectroscopy is a local probe, so an atom is either S or Cl, it cannot be a ⅓S + ⅔Cl composite; while this accounts for the average structure it is meaningless for vibrational spectroscopy. To model the system a fully ordered structure is required. As shown in Fig. 10 ▸, there are two routes to generate an ordered structure, these result in space groups *P*2_1_/*m* and *P*6_3_/*m* respectively.

For each vibrational mode, the factor group splitting will result in a doubling/sextupling of the number of modes. As both *P*2_1_/*m* and *P*6_3_/*m* are centrosymmetric space groups, half the modes will be Raman allowed and infrared forbidden and *vice versa* for the other half. Assuming that the factor group splitting is small in this system, (so the factor group components are nearly degenerate), it follows that the infrared and Raman spectra will be similar for both models. We note that the experimental spectra show that the infrared and Raman modes are largely coincident, justifying this assumption. Note that *all* the modes are allowed in the INS, although the larger cross section of Cl will mean that modes involving Cl motion will be stronger (total scattering cross sections: C = 5.55, ^nat^Cl = 16.85, S = 1.03 barn, 1 barn = 1 × 10^−28^ m^2^).

**Figure 11:**
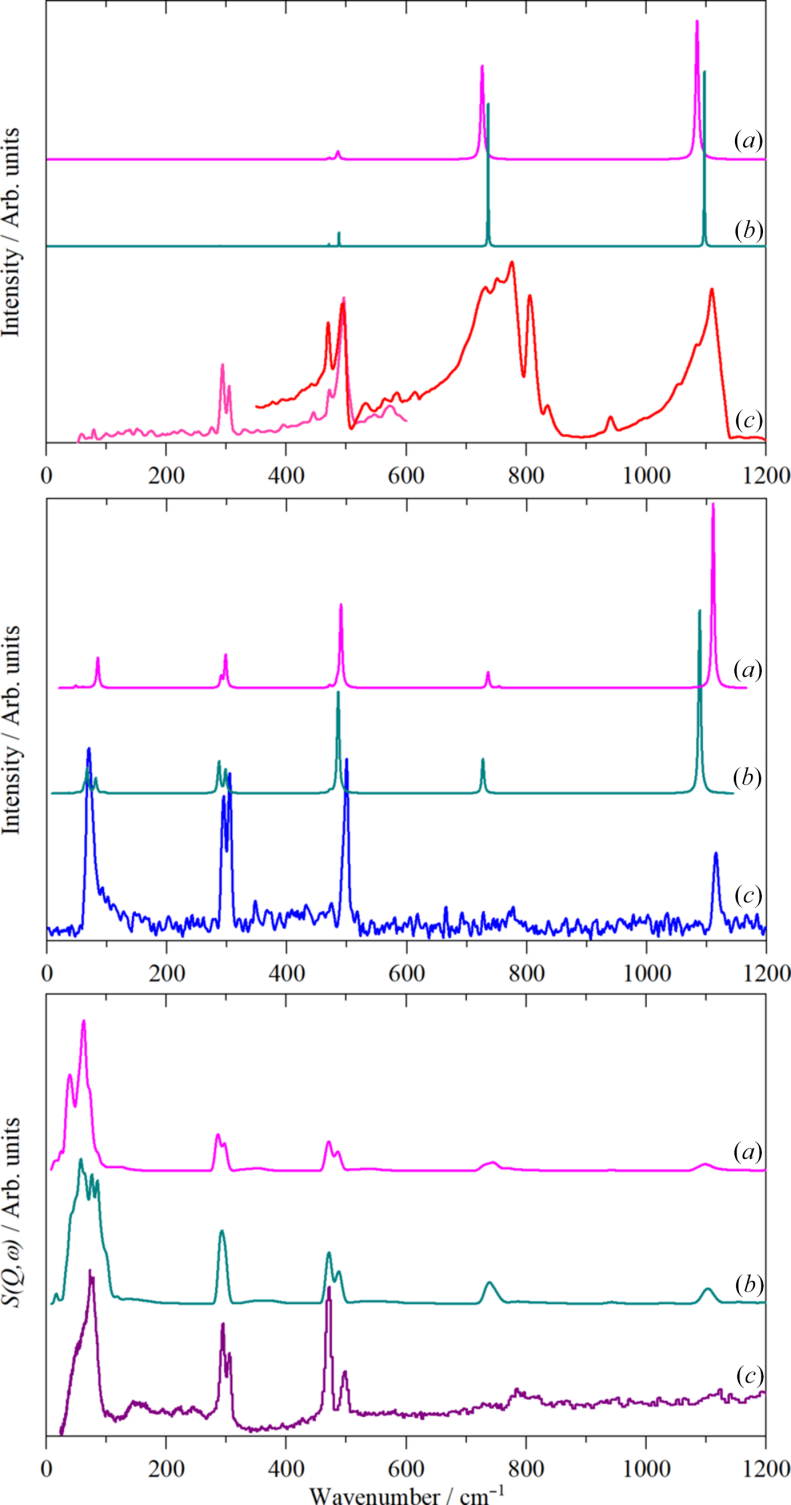
Comparison of observed and calculated solid state spectra of thio­phosgene. Top: infrared; middle: Raman and bottom: INS. In each part (*a*) is the calculated spectrum in *P*2_1_/*m*, (*b*) is that calculated in *P*6_3_/*m* and (*c*) is the experimental data.

The results of the calculations are shown in Fig. 11 ▸. Both structures exhibit all real modes across the entire Brillouin zone, showing that they are dynamically stable (see Fig. S7). The dispersion curves also show that the vibrational dispersion (variation of transition energy with wavevector) is small as shown by the largely horizontal internal modes. It can be seen that for both the Raman and INS spectra, the *P*2_1_/*m* model is inferior to the *P*6_3_/*m* one. This is probably because the larger number of molecules in the latter (*Z* = 2 and 6 respectively) better represents the random arrangement of the molecules in the real (disordered) system. It is noticeable that the two structures predict distinctly different patterns in the external mode region, neither of which matches the experimental data. Clearly, to capture the disorder, a much larger unit cell is required.

The mode visualizations confirm the assignments given in Fig. 9 ▸. The calculations show that for all of the internal modes, the factor group splitting is no more than 20 cm^−1^, so it cannot account for the width and structure of the 800 cm^−1^ band in the infrared spectrum. The ordinate expansion of the INS spectrum in this region shows that there is intensity in the same region. Normal coordinate calculations show that the ^35/37^Cl isotope splitting is less than 10 cm^−1^, so this is unlikely to be the cause. The most likely explanation is that there is Fermi resonance between ν_4_ and the combinations of ν_3_/ν_5_ with ν_2_/ν_6_.

## 3. Conclusion

The melting point of thio­phosgene as determined from differential scanning calorimetry is 231.85 K (−41.3 °C). In its solid state it crystallizes in the hexagonal space group *P*6_3_/*m* with *a* = *b* = 5.9645 (2), *c* = 6.2835 (3) Å. Here, the molecule shows rotational disorder: the sulfur and chlorine atoms form a regular triangle around the central C atom resulting in mixed occupancy of one-third S and two-thirds Cl for the non-C atom positions. As was shown by neutron powder diffraction this disorder is present even at low temperatures. Both *a* and *c* unit-cell parameters increase with increasing temperature between 10 K and 230 K by +1.084 and +5.141%, respectively. From these, the bulk modulus K_0_ was determined for the *a* [12.5 (2) GPa] and *c* [2.45 (2) GPa] unit-cell parameters as well as the unit-cell volume [6.5 (1) GPa].

The IR, Raman and INS spectra for solid state thio­phosgene were collected and interpreted with the aid of quantum chemical calculations. To facilitate the latter, two ordering variants (*P*2_1_/*m* and *P*6_3_/*m* with enlarged unit cell) were artificially created. Comparison of the experimental and calculated spectra allowed for full band assignment. The agreement is better for the model in *P*6_3_/*m*, however, for the external mode region both models predict distinctly different patterns than measured, clearly indicating that for a full description of the disorder, a larger cell must be modelled.

## 4. Related literature

The following references are cited in the supporting information for this article: Clark *et al.* (2005 ▸), Dymkowski *et al.* (2018 ▸), Milman *et al.* (2009 ▸), Parker *et al.* (2014 ▸), Perdew *et al.* (1996 ▸), Pinna *et al.* (2018 ▸), Porezag & Pederson (1996 ▸) and Tkatchenko & Scheffler (2009 ▸).

## Supplementary Material

Crystal structure: contains datablock(s) I, 20K_publ, 30K_publ, 40K_publ, 50K_publ, 60K_publ, 70K_publ, 80K_publ, 90K_publ, 100K_publ, 110K_publ, 120K_publ, 130K_publ, 140K_publ, 150K_publ, 160K_publ, 170K_publ, 180K_publ, 190K_publ, 200K_publ, 210K_publ, 220K_publ, 230K_publ. DOI: 10.1107/S2052520624007583/dq5061sup1.cif

Structure factors: contains datablock(s) I. DOI: 10.1107/S2052520624007583/dq5061Isup2.hkl

Sections S1-S3, Tables S1 and S2, Figs S1-S7. DOI: 10.1107/S2052520624007583/dq5061sup3.pdf

Supporting information file. DOI: 10.1107/S2052520624007583/dq5061230Ksup4.cml

CCDC references: 2375030, 2379235, 2379236, 2379237, 2379238, 2379239, 2379240, 2379241, 2379242, 2379243, 2379244, 2379245, 2379246, 2379247, 2379248, 2379249, 2379250, 2379251, 2379252, 2379253, 2379254, 2379255, 2379256

## Figures and Tables

**Table 1:** Parameters obtained by the fitting of a Debye-type model to the unit-cell parameters of thio­phosgene as a function of temperature [equations (1) and (2)] and potentially meaningful vibrational and elastic parameters The last four rows contain parameters derived from the values in the preceding rows.

	Unit-cell volume	*a* axis	*c* axis
θ_D_ (K)	109 (3)	104 (4)	112 (3)
*V*_0_ (cm^3^ mol^−1^)	57.489 (5)	63.318 (4)	72.96 (1)
*Q* (J cm^−3^)	3.70 (4) × 10^5^	7.9 (1) × 10^5^	1.79 (2) × 10^5^
*b*	5.9 (2)	10.6 (5)	3.23 (6)
			
Debye cut-off (cm^−1^)	76 (2)	72 (3)	78 (2)
V_0_ (Å^3^), *a*_0_, *c*_0_ (Å)	190.93 (2)	5.9466 (1)	6.2344 (3)
K_0_/γ (gPa)	6.5 (1)	12.5 (2)	2.45 (2)
K′_0_	12.8 (3)	22 (1)	7.5 (1)

## References

[bb1] Arnold, O., Bilheux, J. C., Borreguero, J. M., Buts, A., Campbell, S. I., Chapon, L., Doucet, M., Draper, N., Ferraz Leal, R., Gigg, M. A., Lynch, V. E., Markvardsen, A., Mikkelson, D. J., Mikkelson, R. L., Miller, R., Palmen, K., Parker, P., Passos, G., Perring, T. G., Peterson, P. F., Ren, S., Reuter, M. A., Savici, A. T., Taylor, J. W., Taylor, R. J., Tolchenov, R., Zhou, W. & Zikovsky, J. (2014). *Nucl. Instrum. Methods Phys. Res. A*, **764**, 156–166.

[bb2] Billeter, O. & Strohl, A. (1888). *Ber. Dtsch. Chem. Ges.* **21**, 102–110.

[bb4] Burnelle, L. (1956). *J. Chem. Phys.* **24**, 620.

[bb5] Clark, S. J., Segall, M. D., Pickard, C. J., Hasnip, P. J., Probert, M. I. J., Refson, K. & Payne, M. C. (2005). *Z. Kristallogr.* **220**, 567–570.

[bb6] Cochran, W. (1973). *The Dynamics of Atoms in Crystals*. London: Arnold.

[bb7] Coelho, A. A. (2018). *J. Appl. Cryst.* **51**, 210–218.

[bb8] Dymkowski, K., Parker, S. F., Fernandez-Alonso, F. & Mukhopadhyay, S. (2018). *Physica B*, **551**, 443–448.

[bb9] Fortes, A. D. (2019). *Phys. Chem. Chem. Phys.* **21**, 8264–8274. 10.1039/c9cp01234f 30942245

[bb11] Jones, J. I., Kynaston, W. & Hales, J. L. (1957). *J. Chem. Soc.* pp. 614–618.

[bb12] Klason, P. (1887). *Ber. Dtsch. Chem. Ges.* **20**, 2376–2383.

[bb13] Kolbe, H. (1843). *Justus Liebigs Ann. Chem.* **45**, 41–46.

[bb14] Larson, A. C. & Von Dreele, R. B. (2004). *GSAS*. Report LAUR 86-748. Los Alamos National Laboratory, New Mexico, USA. https://subversion.xray.aps.anl.gov/EXPGUI/gsas/all/GSAS%20Manual.pdf.

[bb15] Lowell, R. J. & Jones, E. A. (1960). *J. Mol. Spectrosc.* **4**, 173–189.

[bb16] Milman, V., Perlov, A., Refson, K., Clark, S. J., Gavartin, J. & Winkler, B. (2009). *J. Phys. Condens. Matter*, **21**, 485404. 10.1088/0953-8984/21/48/485404 21832518

[bb17] Nakata, M., Fukuyama, T. & Kuchitsu, K. (1982). *J. Mol. Struct.* **81**, 121–129.

[bb18] Parker, S. F., Fernandez-Alonso, F., Ramirez-Cuesta, A. J., Tomkinson, J., Rudic, S., Pinna, R. S., Gorini, G. & Fernández Castañon, J. (2014). *J. Phys. Conf. Ser.* **554**, 012003.

[bb19] Perdew, J. P., Burke, K. & Ernzerhof, M. (1996). *Phys. Rev. Lett.* **77**, 3865–3868. 10.1103/PhysRevLett.77.3865 10062328

[bb20] Pinna, R. S., Rudić, S., Parker, S. F., Armstrong, J., Zanetti, M., Škoro, G., Waller, S. P., Zacek, D., Smith, C. A., Capstick, M. J., McPhail, D. J., Pooley, D. E., Howells, G. D., Gorini, G. & Fernandez-Alonso, F. (2018). *Nucl. Instrum. Methods Phys. Res. A*, **896**, 68–74.

[bb21] Porezag, D. & Pederson, M. R. (1996). *Phys. Rev. B*, **54**, 7830–7836. 10.1103/physrevb.54.7830 9984457

[bb22] Tambornino, F., Pfeiffer, J. & Fortes, A. D. (2024). Variable-temperature study of phosgene (COCl_2_) and thiophosgene (CSCl_2_). STFC ISIS Neutron and Muon Source. https://doi.org/10.5286/ISIS.E.RB2220167.

[bb23] Tilles, H. (1966). *The Chemistry of Organic Sulfur Compounds*, edited by N. Kharasch & C. Y. Meyers, pp. 311–336. Oxford: Pergamon Press.

[bb24] Tkatchenko, A. & Scheffler, M. (2009). *Phys. Rev. Lett.* **102**, 073005. 10.1103/PhysRevLett.102.073005 19257665

[bb25] Toby, B. H. (2001). *J. Appl. Cryst.* **34**, 210–213.

[bb26] Zaslow, B., Atoji, M. & Lipscomb, W. N. (1952). *Acta Cryst.* **5**, 833–837.

