# Supplementary material for: A comprehensive characterization of thiophosgene in the solid state
Source: Acta Crystallogr B Struct Sci Cryst Eng Mater. 2024 Sep 5;80(Pt 5):495–503. doi: 10.1107/S2052520624007583 (PMC11457100; doi:10.1107/S2052520624007583)
Supplement: Supplementary file 3 [file b-80-00495-sup3.pdf]

Acta Cryst B
STRUCTURAL SCIENCE
CRYSTAL ENGINEERING
MATERIALS

Volume 80 (2024)

Supporting information for article:

A comprehensive characterization of thiophosgene in the solid state

Frank Tambornino, Sven Ringelband, Stewart F. Parker, Christopher M. Howard and Dominic Fortes

S1. Experimental

S1.1. Diffraction

Table S1 Refined lattice parameters from neutron powder diffraction data (and derived axial ratio, *c*/*a*) of thiophosgene as a function of temperature.

T (K)	*a*-axis (Å)	*c*-axis (Å)	V (Å^3)	*c*/*a*
10.00(2)	5.94681(1)	6.23506(1)	190.9590(5)	1.048471(2)
20.01(3)	5.94714(2)	6.23667(3)	191.029(1)	1.048684(6)
29.99(3)	5.94831(2)	6.24141(3)	191.249(1)	1.049275(6)
39.99(3)	5.94993(2)	6.24867(3)	191.576(1)	1.050209(6)
50.00(3)	5.95197(1)	6.25772(2)	191.986(1)	1.051370(3)
59.99(3)	5.95423(2)	6.26813(3)	192.451(1)	1.052719(6)
70.00(3)	5.95667(2)	6.27952(3)	192.959(1)	1.054199(6)
80.00(3)	5.95929(2)	6.29165(3)	193.501(1)	1.055771(7)
90.00(2)	5.96198(2)	6.30443(3)	194.069(1)	1.057440(7)
100.00(2)	5.96491(1)	6.31778(2)	194.672(1)	1.059157(4)
110.00(1)	5.96782(3)	6.33180(4)	195.294(1)	1.060990(7)
120.01(1)	5.97088(2)	6.34639(3)	195.945(1)	1.062891(7)
130.00(1)	5.97392(2)	6.36164(3)	196.616(1)	1.064903(7)
140.00(1)	5.97709(2)	6.37740(3)	197.312(1)	1.066975(7)
150.01(1)	5.98029(1)	6.39382(3)	198.032(1)	1.069149(4)
160.00(1)	5.98355(2)	6.41059(3)	198.768(1)	1.071370(7)
170.00(1)	5.98690(2)	6.42811(3)	199.535(1)	1.073695(7)
180.00(1)	5.99065(2)	6.44691(4)	200.369(1)	1.076163(7)
190.00(1)	5.99449(2)	6.46654(4)	201.237(1)	1.078747(7)
200.00(1)	5.99843(1)	6.48719(2)	202.144(1)	1.081482(4)
210.00(1)	6.00250(3)	6.50883(4)	203.094(1)	1.084354(8)
220.01(1)	6.00678(3)	6.53172(4)	204.099(2)	1.087392(9)
230.00(1)	6.01129(3)	6.55563(5)	205.154(2)	1.09055(1)

Table S2 Derived coefficients of linear (α_1, α_3) and volume (α_V) thermal expansion in thiophosgene as a function of temperature.

T (K)	$\alpha_1 = \alpha_2$ (x10^{-4} K^{-1})	α_3 (x10^{-4} K^{-1})	α_V (x10^{-4} K^{-1})
15.01(2)	0.06(1)	0.26(1)	0.37(1)
25.00(3)	0.20(2)	0.76(1)	1.15(2)
35.0(2)	0.27(2)	1.16(4)	1.71(6)
45.0(2)	0.34(2)	1.45(5)	2.13(7)
55.00(3)	0.38(1)	1.67(1)	2.42(2)
65.00(3)	0.41(2)	1.82(2)	2.64(3)
75.00(3)	0.44(2)	1.93(2)	2.81(3)
85.00(3)	0.45(2)	2.03(2)	2.93(3)
95.00(2)	0.49(2)	2.12(2)	3.10(3)
105.00(2)	0.49(2)	2.22(1)	3.20(2)
115.01(1)	0.51(2)	2.30(2)	3.33(3)
125.01(1)	0.51(2)	2.40(2)	3.42(3)
135.00(1)	0.53(2)	2.48(1)	3.54(3)
145.00(1)	0.54(2)	2.57(1)	3.64(2)
155.01(1)	0.55(2)	2.62(1)	3.71(2)
165.00(1)	0.56(2)	2.73(2)	3.85(3)
175.00(1)	0.63(2)	2.92(2)	4.17(3)
185.00(1)	0.64(2)	3.05(2)	4.33(3)
195.00(1)	0.66(2)	3.19(2)	4.51(3)
205.00(1)	0.68(2)	3.34(1)	4.70(2)
215.00(1)	0.71(2)	3.51(2)	4.94(3)
225.00(1)	0.75(3)	3.66(2)	5.17(3)

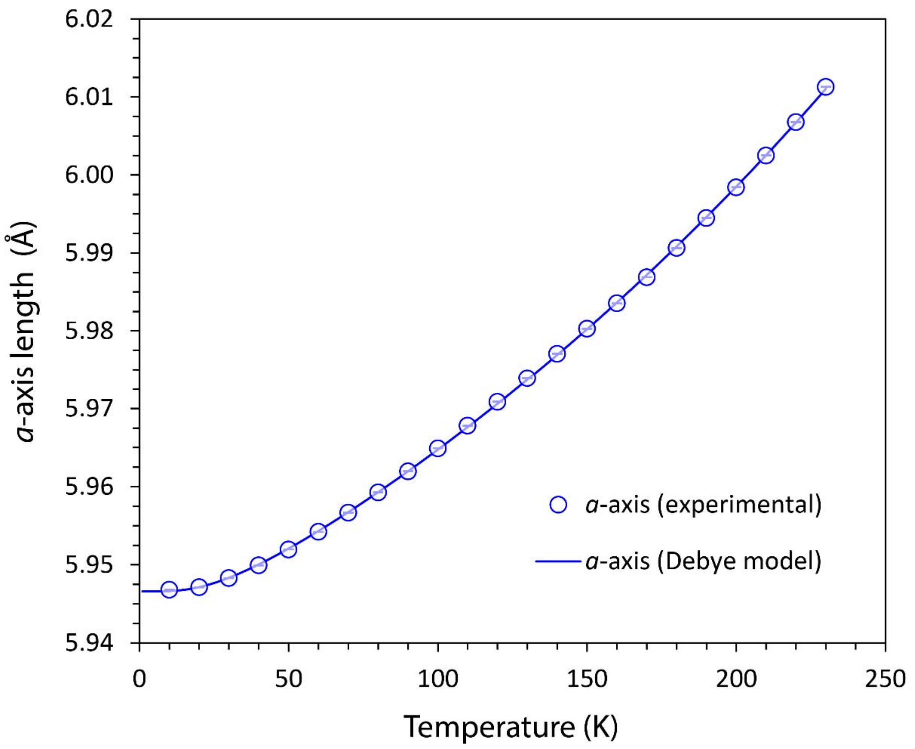

Figure S1 Variation of the *a*-axis length in thiophosgene with temperature. See main text for details of the model fitting.

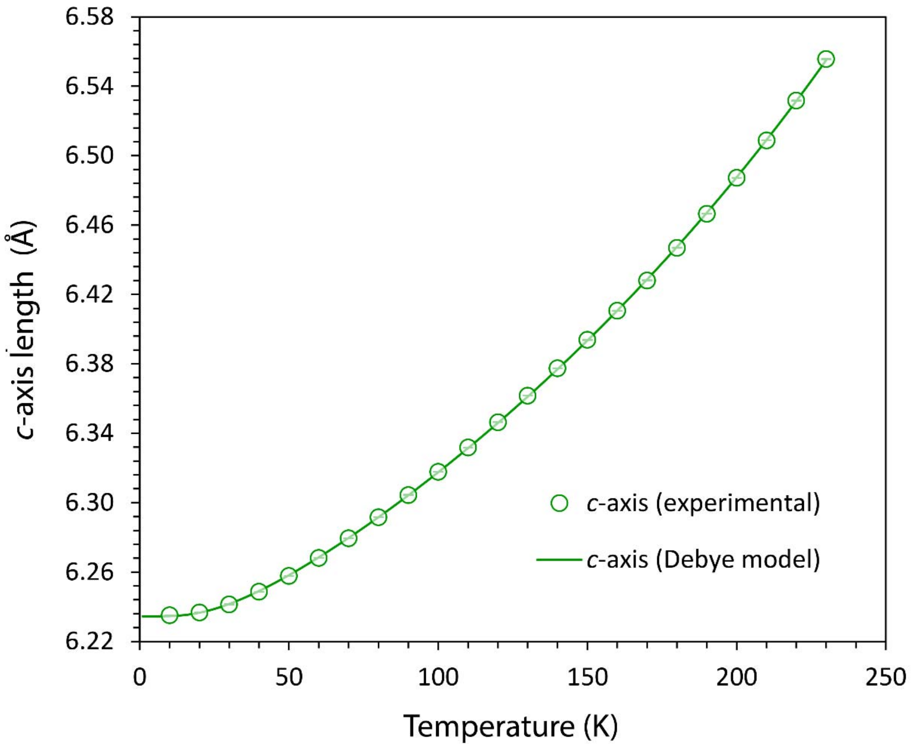

Figure S2 Variation of the *c*-axis length in thiophosgene with temperature. See main text for details of the model fitting.

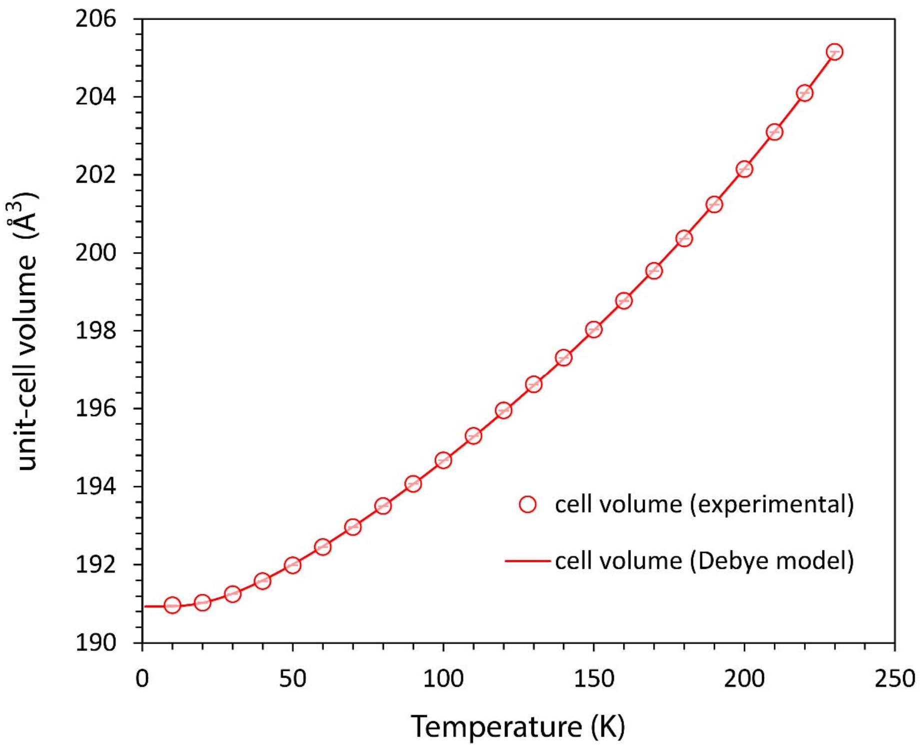

Figure S3 Variation of the unit-cell volume in thiophosgene with temperature. See main text for details of the model fitting.

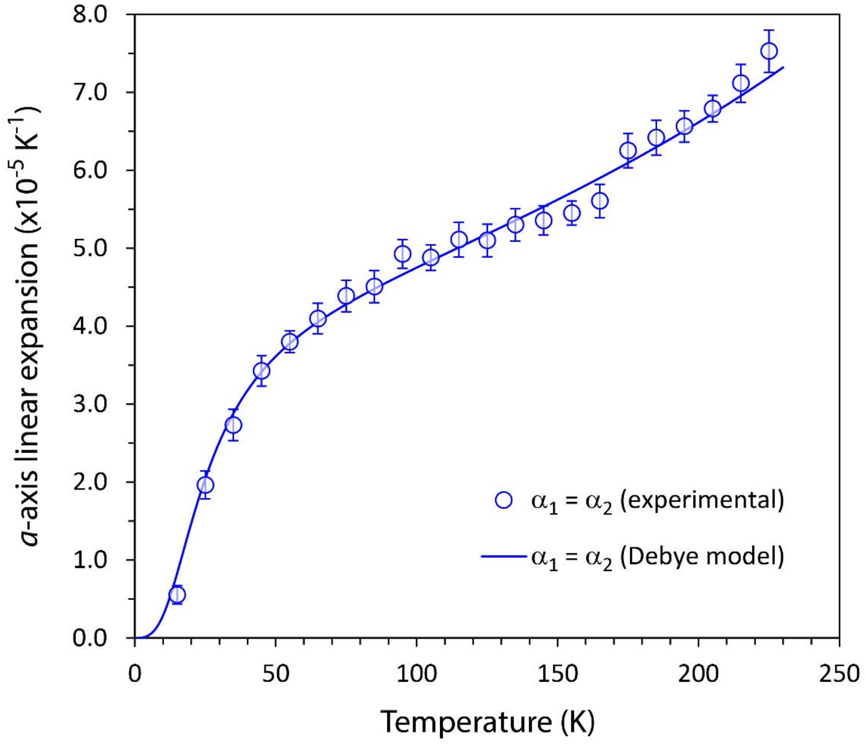

Figure S4 Variation of the linear thermal expansion along the [1000] direction in thiophosgene with temperature. See main text for details of the model fitting.

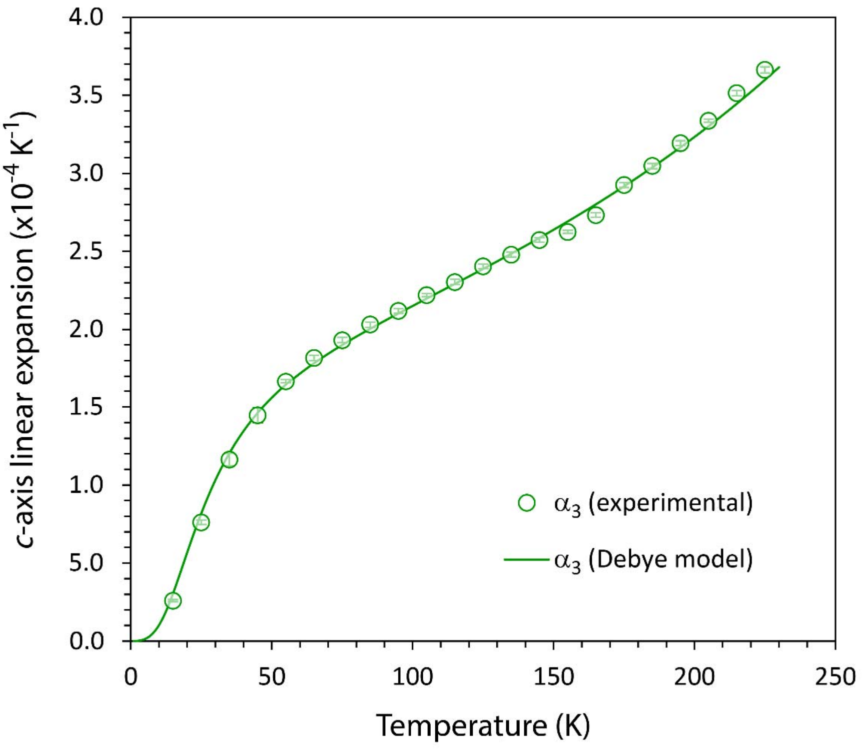

Figure S5 Variation of the linear thermal expansion along the [0001] direction in thiophosgene with temperature. See main text for details of the model fitting.

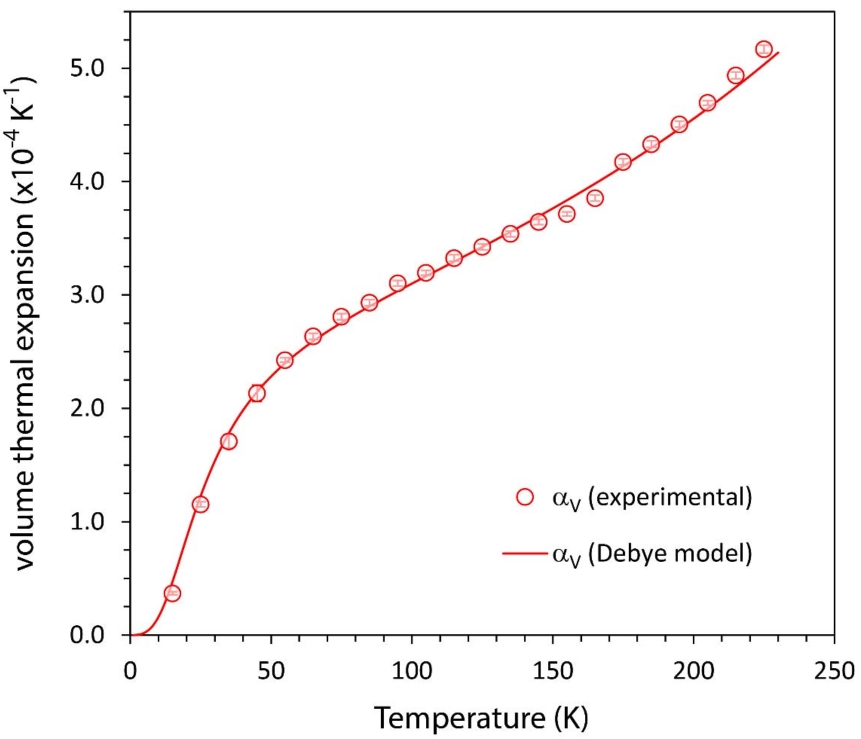

Figure S6 Variation of the volume thermal expansion in thiophosgene with temperature. See main text for details of the model fitting.

S1.2. Vibrational spectroscopy

Room temperature infrared spectra were recorded with a Bruker Vertex70 FTIR spectrometer by attenuated total internal reflection (ATR) using a Bruker Diamond ATR accessory (256 scans, 4 cm^{-1} resolution across a range of 4000 to 50 cm^{-1} with 8 × zerofilling to improve the peak shape). Measurements of the solid were made with a SpecAc Golden Gate low temperature accessory. An extended ATR correction (using the Bruker software) was applied to all measurements to correct for the wavelength-dependent penetration depth. To provide a longer pathlength, some measurements were also made using a transmission cell with polyethylene windows to access the low energy modes.

Raman spectra were recorded with a Bruker FT-Raman spectrometer using 1064 nm excitation (16 scans, 500 mW, 4 cm^{-1} resolution with 8 x zerofilling) of the sample in a quartz cell. Spectra of the solid were recorded by immersing the cell in liquid nitrogen.

Inelastic neutron scattering (INS) spectra were recorded with TOSCA (Parker *et al.*, 2014; Pinna *et al.*, 2018) at ISIS (Chilton, UK; https://www.isis.stfc.ac.uk/Pages/About.aspx) The liquid, ~5 g, was loaded into an indium wire sealed aluminium can and cooled to ~10 K for measurement.

S1.3. Computational methods

Dispersion corrected periodic density functional theory (DFT-D) calculations were carried out with CASTEP (versions 21.21 and 23.1) (Clark *et al.*, 2005). On-the-fly generated norm conserving pseudopotentials with a plane-wave cut-off of 720 eV were used with the PBE(Perdew *et al.*, 1996) functional with the Tkatchenko-Scheffler (Tkatchenko & Scheffler, 2009) dispersion correction scheme within the generalized gradient approximation (GGA). The Brillouin zone sampling of electronic states was performed with a Monkhorst-Pack grid ($P2_1/m$: 8 × 6 × 8, 96 *k*-points; $P6_3/m$: 4 × 6 × 8, 48 *k*-points). The equilibrium structure, an essential prerequisite for lattice dynamics calculations, was obtained by BFGS geometry optimization. Phonon frequencies were obtained by diagonalization of the dynamical matrix, computed using density-functional perturbation theory (Milman *et al.*, 2009) to compute the dielectric response and the Born effective charges, and, from these, the mode oscillator strength tensor and infrared absorptivity were calculated. Raman intensities were calculated by a finite displacement method (Porezag & Pederson, 1996). In addition to the calculation of transition energies at zero wavevector, for some systems, phonon dispersion was also calculated along high symmetry directions throughout the Brillouin zone. For this purpose, dynamical matrices were computed on a regular grid of wavevectors throughout the Brillouin zone, and Fourier interpolation was used to extend the computed grid to the desired fine set of points along the high-symmetry paths. The atomic displacements in each mode, that are part of

the CASTEP output, enable visualization of the modes in Materials Studio (BIOVIA; https://www.3dsbiovia.com/products/collaborative-science/biovia-materials-studio/) to aid assignments and are also all that is required to generate the INS spectrum using the program AbINS (Dymkowski *et al.*, 2018). It is emphasised that, for the calculated spectra and dispersion curves shown, the transition energies have *not* been scaled.

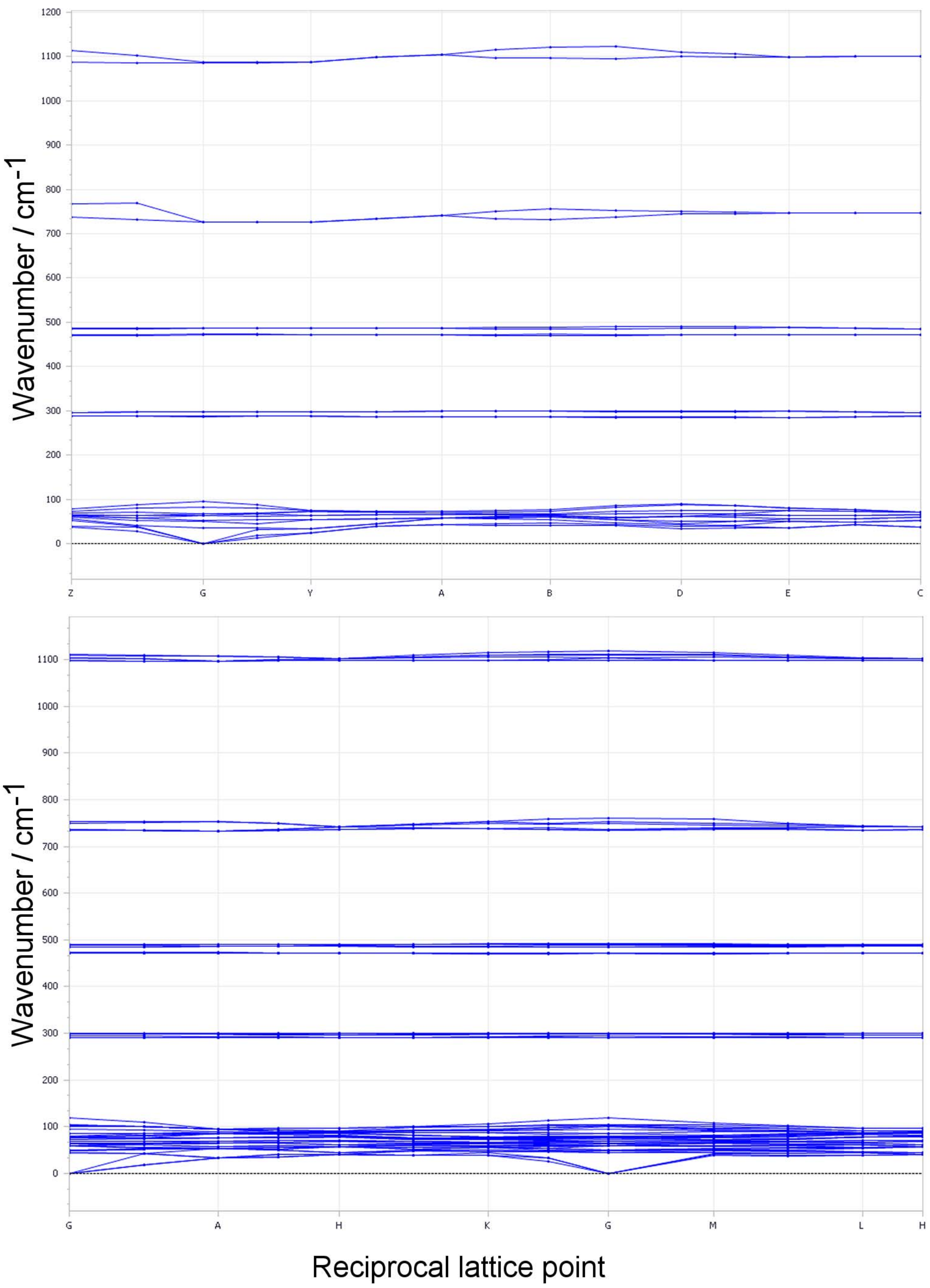

Figure S7 Dispersion curves of thiophosgene in: top $P2_1/m$ and bottom $P6_3/m$.
